# Melatonin inhibits 17β-estradiol-induced migration, invasion and epithelial-mesenchymal transition in normal and endometriotic endometrial epithelial cells

**DOI:** 10.1186/s12958-018-0375-5

**Published:** 2018-06-23

**Authors:** Shasha Qi, Lei Yan, Zhao Liu, Yu-lan Mu, Mingjiang Li, Xingbo Zhao, Zi-Jiang Chen, Hui Zhang

**Affiliations:** 1 Center for Reproductive Medicine, Shandong Provincial Hospital Affiliated to Shandong University, Jinan, 250021 People’s Republic of China; 2 National Research Center for Assisted Reproductive Technology and Reproductive Genetics, Jinan, 250021 People’s Republic of China; 3 The Key laboratory for Reproductive Endocrinology, Shandong University, Ministry of Education, Jinan, 250021 People’s Republic of China; 4 Department of Urology, Qilu Hospital of Shandong University, 107 Wenhua Xi Road, Jinan, 250012 People’s Republic of China; 5 Department of Obstetrics and Gynecology, Shandong Provincial Hospital Affiliated to Shandong University, 324 Jingwu Road, Jinan, 250021 People’s Republic of China; 6 Shanghai Key Laboratory for Assisted Reproduction and Reproductive Genetics, Shanghai, 200030 People’s Republic of China; 7 Center for Reproductive Medicine, Ren Ji Hospital, School of Medicine, Shanghai Jiao Tong University, Shanghai, 200030 People’s Republic of China

**Keywords:** Endometrial epithelial cells, Melatonin, 17β-estradiol, Migration and invasion, Epithelial-mesenchymal transition

## Abstract

**Background:**

Melatonin is a potential therapeutic agent for endometriosis, but its molecular mechanism is unclear. Here, we investigated the effect of melatonin on the epithelial-mesenchymal transition (EMT) in endometriotic endometrial epithelial cells and explored the pathway that might be involved.

**Methods:**

This hospital-based study included 60 women of reproductive age using the endometrium for immunohistochemistry, 6 women of reproductive age undergoing bilateral tubal ligation and 6 patients with endometriosis for isolation of endometrial epithelial cells or subsequent analysis, respectively. We examined the expression of Notch1/Numb signaling and EMT markers by immunohistochemistry analysis and western blot analysis, the invasion and migration of endometrial epithelial cells by transwell assays, and the cell proliferation by CCK8 assays.

**Results:**

Compared with normal endometrium, the endometriotic eutopic endometrium showed increased expression of Notch1, Slug, Snail, and N-cadherin, and decreased expression of E-cadherin and Numb. Melatonin or Notch inhibition by specific inhibitor blocked 17β-estradiol-induced cell proliferation, invasion, migration and EMT-related markers in both normal and endometriotic epithelial cells.

**Conclusions:**

Our data suggest that aberrant expression of Notch1/Numb signaling and the EMT is present in endometriotic endometrium. Melatonin may block 17β-estradiol-induced migration, invasion and EMT in normal and endometriotic epithelial cells by upregulating Numb expression and decreasing the activity of the Notch signaling pathway.

## Background

Endometriosis is a chronic disease that affects approximately 10% of reproductive-age women and is characterized by chronic pelvic pain and infertility [1]. Endometriosis is a benign disease, but its tendency to progression and recurrence causes disability and distress [2]. In recent decades, much effort has been focused on developing new drugs to relieve the clinical symptoms and prevent the recurrence of the disease.

Epithelial-mesenchymal transition (EMT), which is characterized by an increased rate of cellular migration, invasion properties and increased resistance to apoptosis, is also considered essential for the formation and progression of endometriosis [3–5]. Key transcription factors including Snail, Slug and Twist drive the EMT process, which along with cleaved and then degraded E-cadherin and increased expression of mesenchymal-related proteins, including N-cadherin, Vimentin [6]. Snail also represses E-cadherin transcription by binding to the E-box site in the promoter of E-cadherin [7]. In previous studies, we found that the expression of both Notch1, a key signaling factor involved in EMT regulation, and EMT-related proteins was upregulated in the ectopic endometrium of adenomyosis compared with those in normal endometrium [8].

Melatonin (N-acetyl-5-methoxy-tryptamine), a scavenger of free radicals and a broad-spectrum antioxidant, is the main pineal hormone synthesized from tryptophan in response to darkness [9]. A series of studies have shown that melatonin has a potential therapeutic effect on endometriosis [10–16]. In experimental rat models, melatonin causes the regression and atrophy of endometriotic lesions [11], and the combination of letrozole and melatonin causes a significant regression in lesion volumes but not in the histopathological scores of endometriotic lesions [15]. Higher doses of melatonin treatment have been reported to be more effective in inducing the regression of implants and in improving histologic scores [10]. Moreover, compared with letrozole, melatonin causes a more pronounced regression of endometriotic foci and lower recurrence [16]. It was reported that pinealectomy increased the progression of endometriosis explants, and that melatonin reversed the effects of pinealectomy [12]. Melatonin is effective in treating experimental endometriosis induced by implanting human endometriotic cells in SCID mice [14]. Kocadal et al. [13] reported that melatonin caused a regression of endometriotic lesions and an improvement in their histopathological scores in an oophorectomized rat endometriosis model.

Melatonin has been proved to be involved in the modulation of EMT. Lipopolysaccharide- induced EMT was inhibited by melatonin in peritoneal mesothelial cells via the inactivation of the Toll-like receptor (TLR) 4/c-Jun N-terminal kinase and TLR4/NFκB-Snail signaling pathways [17]. It has also been reported that melatonin inhibits TGFβ1-induced EMT in human lung alveolar epithelial cells [18]. In the process of bleomycin-induced pulmonary fibrosis, melatonin significantly attenuated endoplasmic reticulum stress-mediated EMT [19]. However, the effects of melatonin on the EMT in endometriosis are unclear.

In the current study, we assumed that melatonin might be involved in the EMT regulation of endometriosis. We investigated the effect of melatonin on the migration, invasion and EMT of normal and endometriotic epithelial cells, and explored the possible signaling pathways that might be involved.

## Methods

### Materials

CollagenaseΙA, trypsin, Melatonin and Matrigel were obtained from Sigma-Aldrich (St. Louis, MO, USA). Penicillin, DMEM/F12 (1:1) media were obtained from HyClone (Logan, Utah, USA). Charcoal-stripped fetal bovine serum (FBS) was obtained from GIBCO (Invitrogen, NY, USA). Rabbit anti-human E-Cadherin, N-Cadherin and Vimentin primary antibodies were obtained from Abcam (Cambridge, MA, USA). Rabbit anti-human Notch, Numb, Slug and Snail primary antibodies were obtained from Cell Signaling Technology (Danvers, MA, USA) for western blot and obtained from Abcam (Cambridge, MA, USA) for immunohistochemistry. Mouse anti-human β-Actin primary antibody and Goat anti-rabbit and Goat anti-mouse HRP-conjugated secondary antibodies were obtained from ZSGB-BIO (Beijing, China). Mammalian Cell Protein Extraction Kit was purchased from Beyotime (Shanghai, China). ECL Plus Western Blotting Detection System was obtained from Millipore Corporation (Billerica, MA, USA).

### Tissue collection and immunohistochemistry analysis

Normal endometria were obtained from 30 women of reproductive age undergoing bilateral tubal ligation (proliferative phase: *n* = 15; secretory phase: *n* = 15). Endometriotic eutopic endometria were obtained from 30 patients with endometriosis undergoing hysterectomy or subtotal hysterectomy (proliferative phase: *n* = 15; secretory phase: *n* = 15). Normal and endometriotic endometrial tissues were collected during the surgery. The diagnosis of endometriosis was confirmed by histological examination. No patients had received any hormonal therapy in the 3 months prior to their surgery. Immunohistochemistry analysis was performed on normal and endometriotic endometria as previously reported [8]. The primary antibodies used in this study included Rabbit anti-human E-Cadherin(4 μg/ml), N-Cadherin(2 μg/ml), Notch1(400 μg/ml), Numb(7 μg/ml), Slug(7 μg/ml), and Snail(5 μg/ml) antibodies. Immunostaining was expressed as the immunoscore, i.e., the H-score, which was a semi quantitative product of the quantity score and staining intensity. The quantity score was estimated as reported previously [8].

### Tissue collection and cells culture

Eutopic endometria were obtained from 6 patients with endometriosis. Normal endometria obtained from 6 women of reproductive age undergoing bilateral tubal ligation were used as controls. All participants had regular menstrual cycles and had not received any hormonal therapy in the 3 months prior to their surgery. Diagnoses were confirmed by histological examination. Endometriotic eutopic epithelial cells (EEC) and normal endometrial epithelial cells (NEC) were isolated from fresh tissues. Isolation and culture of endometrial cells were conducted as reported previously [20]. Briefly, specimens obtained during surgery were placed immediately in ice-cold sterile PBS and transported to the laboratory. Tissues were washed twice with sterile PBS to remove the blood, minced into small pieces, and incubated with 0.25% collagenase type IA in a shaking water bath for 1 h at 37 °C. The collagenase activity was terminated by adding three volumes of pre-warmed medium containing 10% FBS. The cell suspension was sequentially filtered through a 154 μm monofilament nylon mesh and then through a 38.5 μm monofilament nylon mesh. The 38.5 μm monofilament was washed thoroughly upside down with medium to obtain epithelial cells. The resulting cell suspension was collected and centrifuged at 110 g for 10 min. The pellet was re-suspended in DMEM/F12 (1:1) medium containing 10% FBS and was incubated in cell culture dishes for 2 h at 37 °C in 95% air and 5% CO_2_. The medium was then replaced with fresh medium; non-attached cells were discarded, and the attached epithelial cells were cultured further. The culture medium was changed every 2–3 days. The cultured cells were characterized by immunocytochemical staining with mouse anti-human Cytokeratin antibodies, the purified epithelial cells were positive for Cytokeratin [21]. The purity for cells of 1 passage was more than 98%. The primary cultured cells were used in the western blot and morphology experiments. The cells of passage 1 were used in the CCK-8 and transwell experiments.

### Cell treatments

The cells were initially cultured without any estradiol, and were cultured about 48 h until passage. The culture media was changed every 2 to 3 days. The cells were about 50% confluent before treatment. Melatonin was dissolved in ethanol at a stock concentration of 100 mM and was stored at − 20 °C. 17β-estradiol was dissolved in ethanol at a stock concentration of 10 mM and stored at − 20 °C. DAPT, a specific Notch inhibitor, was dissolved in DMSO at a stock concentration of 10 mM and stored at − 20 °C. The concentrations chosen for the used treatments were determined according to published literatures. The cells were treated with 1 mM melatonin [22, 23], 10 μM DAPT [24, 25] or/and 10 nM 17β-estradiol [26, 27]. Mock treatments with an identical volume of ethanol or DMSO were used as controls.

### Transwell assays

The transwell assays were performed using 24-well plates with 8-μm pore size inserts (Corning Life Sciences, NY, USA) according to the manufacturer’s instructions. The cells were treated with various agents at the indicated concentration for 48 h before they were seeded into the inserts. The migration and invasion assays were performed as reported previously [6].

In the migration assay, the cells (normal and endometriotic epithelial cells 10^5^cells/well) were added to the upper chamber in 200 μL of serum-free DMEM medium and were allowed to migrate to the bottom compartment, which contained DMEM medium with 10% FBS, for 24 h. Then, the non-migrated cells were wiped off with a cotton swab.

For the invasion assay, Matrigel (1 mg/ml, BD Biosciences) was prepared in serum-free cold cell culture medium, placed in the upper chamber, and incubated for 5 h at 37 °C. Next, the cells (normal and endometriotic epithelial cells 2 × 10^5^cells/well) were placed in the upper chamber of each insert in 200 μL of serum-free medium, and were allowed to invade to the bottom compartment, which contained medium with 10% FBS, for 36 h. Then, the non-invaded cells were wiped off with a cotton swab.

For quantification, transwell filters were fixed in 4% paraformaldehyde for 15 min, stained with haematoxylin for 15 min, and mounted on a glass slide. The results were expressed as the number of cells migrated per field, as viewed under a microscope (× 200 magnification), and the numbers of cells in three randomly selected fields were counted. All experiments were performed three times.

### Cell proliferation assay

Cell proliferation was assessed using the Cell Counting Kit (CCK)-8 (Dojindo, Japan). Briefly, the cells (normal and endometriotic epithelial cells 8 × 10^3^ cells /well) were plated on 96-well plates in 100 μL of medium and were allowed to attach overnight for 24 h. Then, they were exposed to the indicated concentrations of melatonin and DAPT, with or without 17β-estradiol, and were cultured for an additional 0, 24, 48 and 72 h, respectively. After treatments, 10 μL of CCK-8 reagent was added to each well, and the plates were incubated at 37 °C for 4 h. The cell confluence was about 50–60% confluence at 0 h, and allowed to grow to about 90% confluence at 72 h. The optical density (OD) at 450 nm was measured in each well using a microplate reader. The measurements were performed at the density of 90% confluence approximately. The data were shown as fold change of control (Day 0). The experiments were repeated three times, and each assay was performed in triplicate.

### Total protein extraction and western blot analysis

Cells were harvested by trypsinization and centrifugation. Total protein was extracted, and western blot analysis was performed as reported previously [20]. 50 μg of total protein extracted from cells after 72 h treatments was applied to a 10% polyacrylamide gel, and PageRuler Prestained Protein Ladder (Fermentas) was used as the size marker. After the proteins were transferred to membranes and the membranes were blocked, the membranes were incubated overnight at 4 °C with the primary antibodies. The primary antibodies used in this study included Rabbit anti-human E-Cadherin (0.3 μg/ml), N-Cadherin(1 μg/ml), Vimentin(0.2 μg/ml), Notch1(0.85 μg/ml), Numb(0.26 μg/ml), Slug(0.25 μg/ml), Snail(0.2 μg/ml) and β-actin (0.5 μg/ml). After incubation with the Goat anti-rabbit HRP-conjugated secondary antibody (0.1 μg/ml) for 1 h at room temperature, the protein bands were detected using the ECL detection system (BD Biosciences). β-actin was used as the loading control.

### Statistical analysis

The statistical analyses were performed using SPSS 19.0 (SPSS, Chicago, USA). The values are expressed as the means ± SD. The differences between the two groups were determined by one-way ANOVA. A *p* value < 0.05 was considered statistically significant.

## Results

### Aberrant expression of notch/numb signaling and EMT markers in endometriotic endometrium

**Fig. 1:**
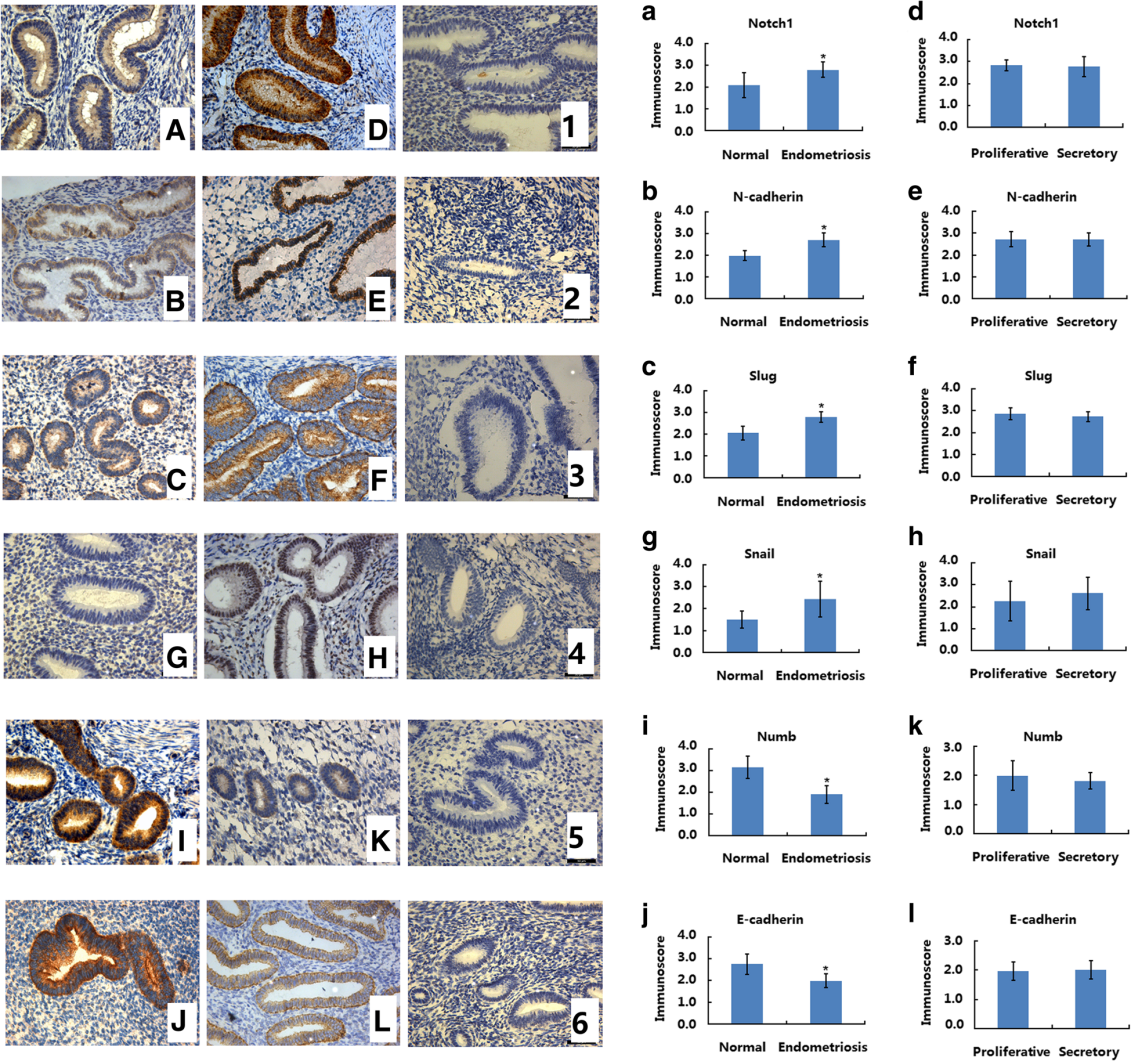
Aberrant expressions of Notch1/Numb signaling and EMT markers in endometrium of endometriosis. **A, B, C, G, I, J**: The expression of Notch1, N-Cadherin, Slug, Snail, Numb and E-Cadherin in normal endometrium (*n* = 15); **D, E, F, H, K, L**: The expression of Notch1, N-Cadherin, Slug, Snail, Numb and E-Cadherin in eutopic endometria of endometriosis (*n* = 15); **a, b, c, g, i, j**: Immunoscore of Notch1, N-Cadherin, Slug, Snail, Numb and E-Cadherin in normal endometrium (*n* = 30) and eutopic endometria of endometriosis (*n* = 30); **d, e, f, h, k, l**: Immunoscore of Notch1, N-Cadherin, Slug, Snail, Numb and E-Cadherin in the proliferative phases (*n* = 15) and secretory phases (*n* = 15) of endometriotic endometria; **1,2,3,4,5,6:** negative controls. Magnification: × 200. *p < 0.05

The expression of Notch/Numb signaling and EMT markers in normal endometria and in endometriotic eutopic endometria were determined by immunohistochemical analysis. We take Notch1 as the representative of Notch family. As shown in Fig. 1, in normal endometria, the staining of Notch1 (NICD) (Fig. 1A), N-cadherin (Fig. 1B), and Slug (Fig. 1C) were weakly positive or positive and were concentrated in the cytoplasm of endometrial epithelial cells. In stromal cells, the immunostainings of Notch1, N-cadherin, and Slug were very weak. In endometriotic eutopic endometria, the immunostaining of Notch1 (Fig. 1D), N-cadherin (Fig. 1E), and Slug (Fig. 1F) was strongly positive and was restricted to the cytoplasm of epithelial cells, whereas weak immunostaining patterns were observed in stromal cells. Endometriotic eutopic endometria showed significantly increased Notch1 (Fig. 1a, *p*< 0.05), N-cadherin (Fig. 1b, *p* < 0.05), and Slug (Fig. 1c, *p* < 0.05) expression levels compared to normal endometria. No significant differences in Notch1 (Fig. 1d, *p* > 0.05), N-cadherin (Fig. 1e, *p* > 0.05), or Slug (Fig. 1f, *p* > 0.05) expression were observed between endometriotic endometria in the proliferative and secretory phases.

In normal endometria, the staining of Snail was weakly positive or positive and was restricted to the nucleus of endometrial epithelial cells (Fig. 1G). In stromal cells, the immunostaining of Snail was very weak. In endometriotic eutopic endometria, the immunostaining of Snail was strongly positive and was restricted to the nucleus of epithelial cells (Fig. 1H), whereas weak immunostaining was observed in stromal cells. Endometriotic eutopic endometria showed significantly increased Snail expression compared to normal endometria (Fig. 1g, *p* < 0.05). No significant difference of Snail expression was observed between endometriotic endometria in the proliferative and secretory phases (Fig. 1h, *p* > 0.05).

In normal endometria, the immunostaining of Numb (Fig. 1I) and E-cadherin (Fig. 1J) was strongly positive, and the staining was concentrated in the cytoplasm of endometrial epithelial cells. In stromal cells, the immunostaining of Numb and E-cadherin was very weak. In endometriotic eutopic endometria, the immunostaining of Numb (Fig. 1K) and E-cadherin (Fig. 1L) was weakly positive and was restricted to the cytoplasm of epithelial cells. Endometriotic eutopic endometria showed significantly decreased Numb (Fig. 1i, *p* < 0.01) and E-cadherin (Fig. 1j, *p* < 0.01) expression compared to normal endometria. No significant difference in Numb (Fig. 1k, *p* > 0.05) and E-cadherin (Fig. 1l, *p* > 0.05) expression was observed between endometriotic endometria in the proliferative and secretory phases.

### Melatonin abolished 17β-estradiol-induced proliferation in normal and endometriotic epithelial cells

**Fig. 2:**
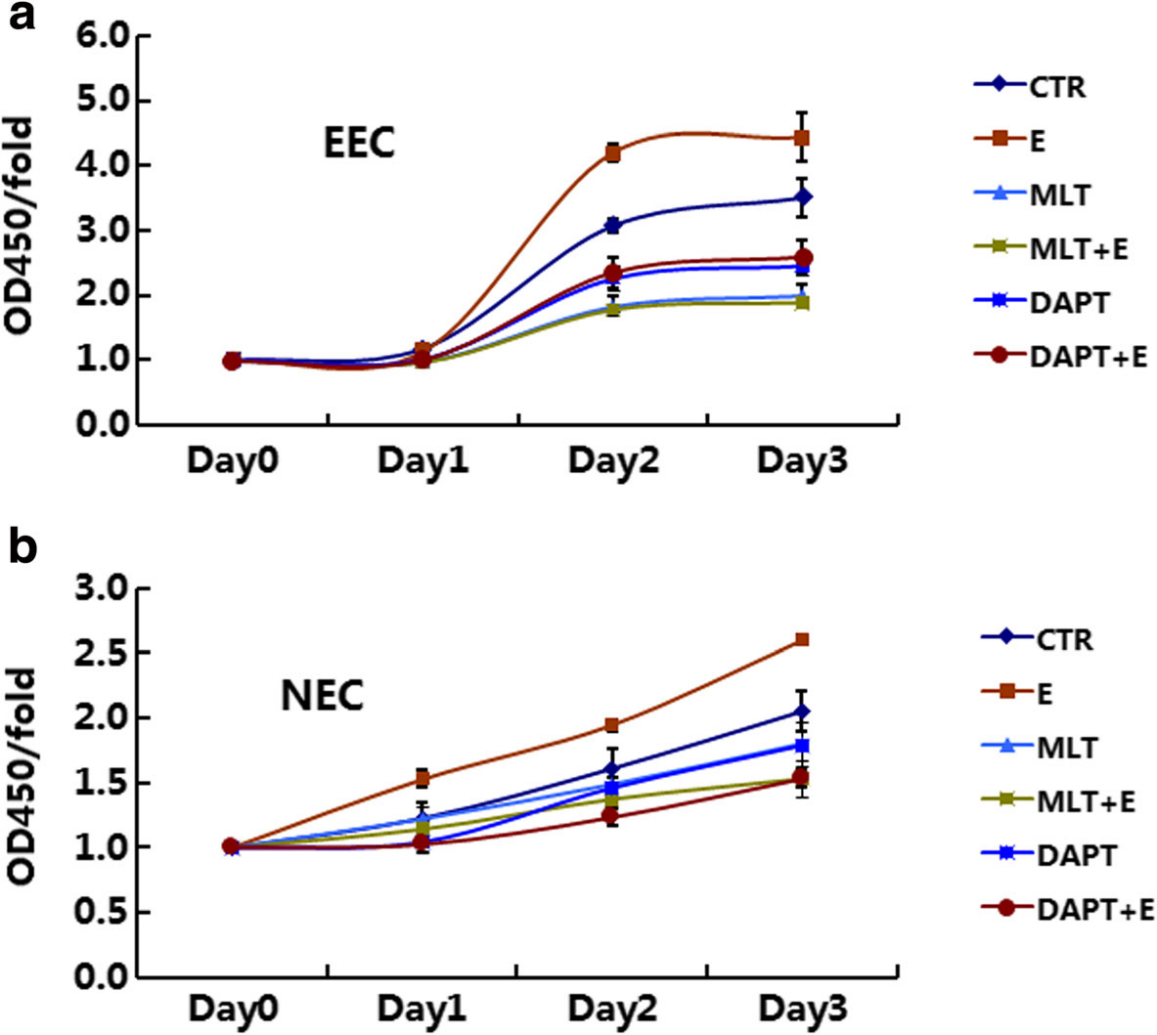
Melatonin abolishes 17β-estradiol-induced proliferation in EEC and NEC. **a**: EEC were treated with MLT, DAPT, or E2 with or without MLT/DAPT, cell numbers were measured by CCK-8 assays at the indicated times. **b**: NEC were treated with MLT, DAPT, or E2 with or without MLT/DAPT, cell numbers were measured by CCK-8 assays at indicated times. Data are presented as the mean ± SD. E2: 17β-estradiol; MLT: melatonin

CCK-8 assays were performed to determine the proliferation of EEC and NEC. 17β-estradiol significantly increased the growth of EEC and NEC on days 2–3 (Fig. 2a, b, *p* < 0.05). DAPT, a specific inhibitor of Notch signaling, significantly decreased the growth of both EEC and NEC (Fig. 2a, b, *p* < 0.05). DAPT also abolished 17β-estradiol-induced cell growth (Fig. 2a, b, *p* < 0.05).

In CCK-8 assays, melatonin significantly decreased the growth of EEC and NEC at day 3 (Fig. 2a, b, *p* < 0.05). Melatonin also abolished 17β-estradiol-induced cell growth in both cells (Fig. 2a, b, *p* < 0.05).

### Melatonin abolished 17β-estradiol-induced migration and invasion in normal and endometriotic epithelial cells

**Fig. 3:**
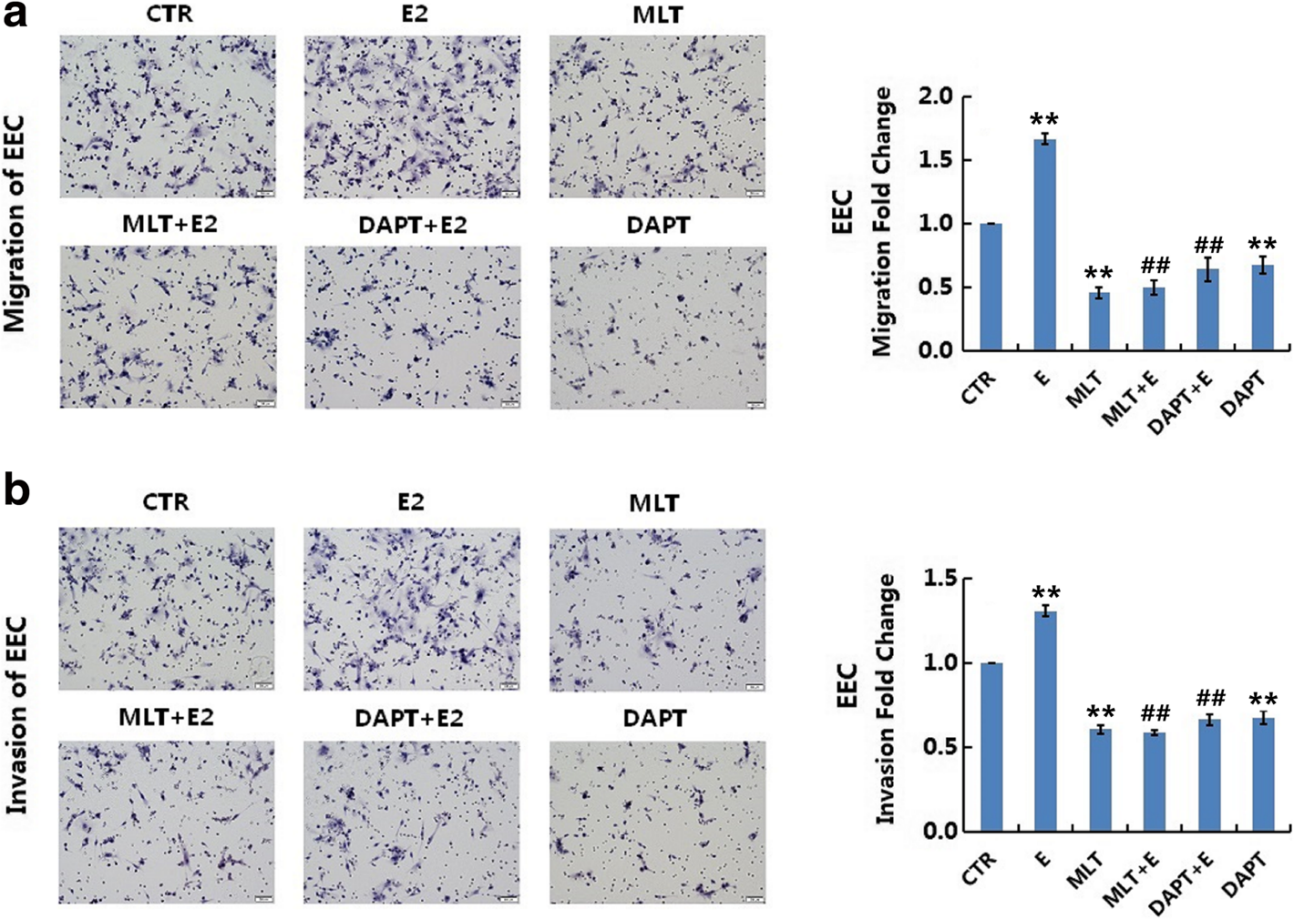
Melatonin abolishes 17β-estradiol-induced migration and invasion in EEC. **a**: Transwell migration assays of EEC after treatment. EEC were treated with MLT, DAPT, or E2 with or without MLT/DAPT **b**: Transwell invasion assays of EEC after treatment. Representative images were obtained at 200× magnification. Graphs show the relative number of migrating and invading cells for each treatment group (averaged across four random images). Scale bar: 50 μm. Data are presented as the mean ± SD. E2: 17β-estradiol; MLT: melatonin. *p < 0.05, ** *p* < 0.01 vs. untreated cells. #*p* < 0.05, ##*p* < 0.01 vs. E2-treated cells

**Fig. 4:**
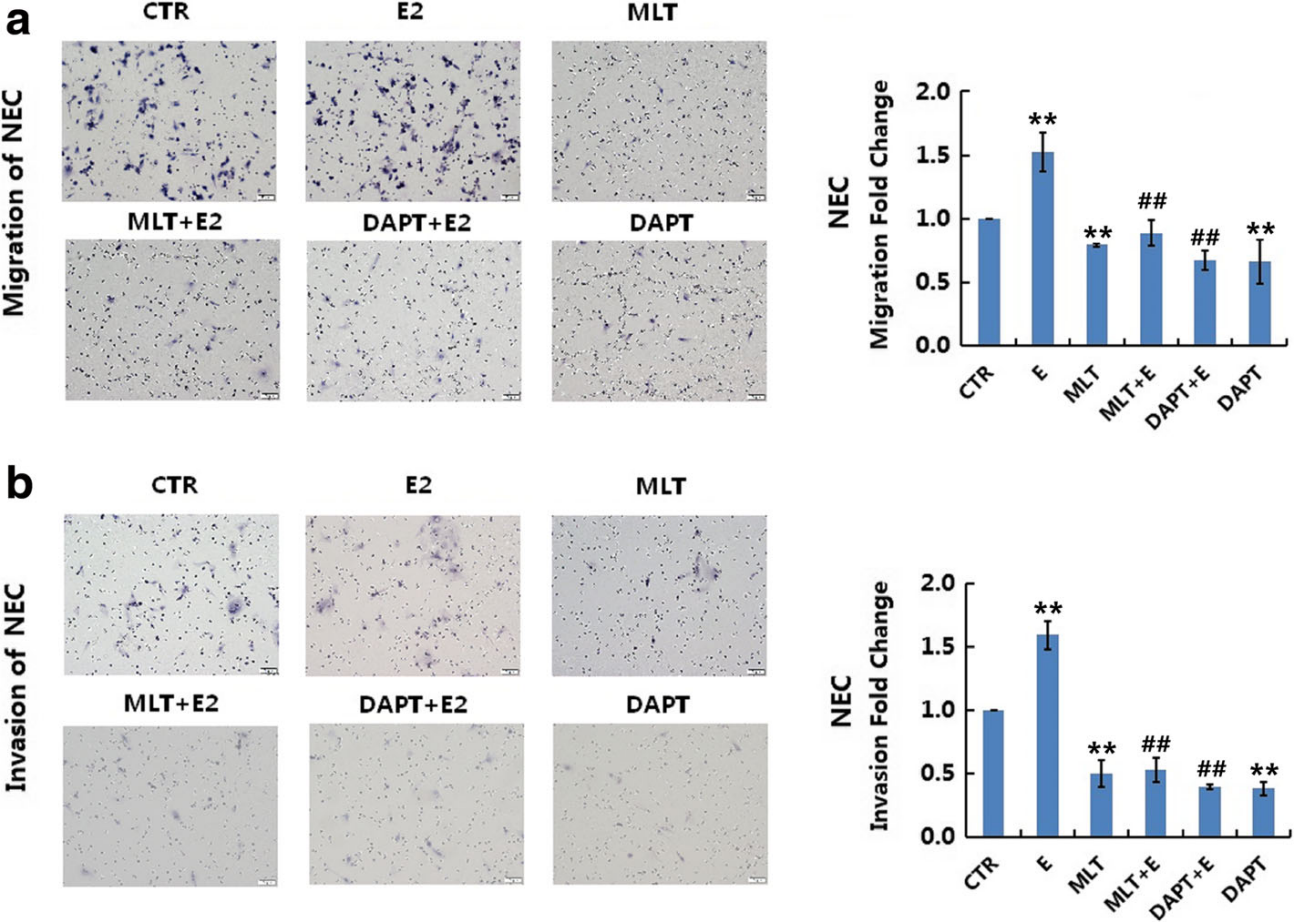
Melatonin abolishes 17β-estradiol-induced migration and invasion in NEC. **a**: Transwell migration assays of NEC after treatment. NEC were treated with MLT, DAPT, or E2 with or without MLT/DAPT **b**: Transwell invasion assays of NEC after treatment. Representative images were obtained at 200× magnification. Graphs show the relative number of migrating and invading cells for each treatment group (averaged across four random images). Scale bar: 50 μm. Data are presented as the mean ± SD. E2: 17β-estradiol; MLT: melatonin. *p < 0.05, ** *p* < 0.01 vs. untreated cells. #*p* < 0.05, ##*p* < 0.01 vs. E2-treated cells

The migration and invasion of EEC (Fig. 3) and NEC (Fig. 4) were determined using transwell assays. In migration assays, 17β-estradiol significantly increased the migration of EEC (*p* < 0.01) and NEC (*p* < 0.01) after treatment for 24 h. DAPT significantly decreased the migration of EEC (*p* < 0.05) and NEC (p < 0.05). DAPT also abolished 17β-estradiol-induced cell migration (*p* < 0.01). Similar data were obtained in invasion assays. 17β-estradiol significantly increased the invasion of EEC (*p* < 0.01) and NEC (p < 0.01) after treatment for 36 h, whereas DAPT significantly decreased the invasion and 17β-estradiol-induced invasion in EEC (*p* < 0.01) and NEC (p < 0.01).

In migration and invasion assays, melatonin significantly decreased the migration and invasion of EEC (Fig. 3, *p* < 0.05) and NEC (Fig. 4, *p* < 0.05). Melatonin also abolished 17β-estradiol-induced cell migration and invasion (*p* < 0.05).

### Melatonin abolished 17β-estradiol-induced EMT in normal and endometriotic epithelial cells

**Fig. 5:**
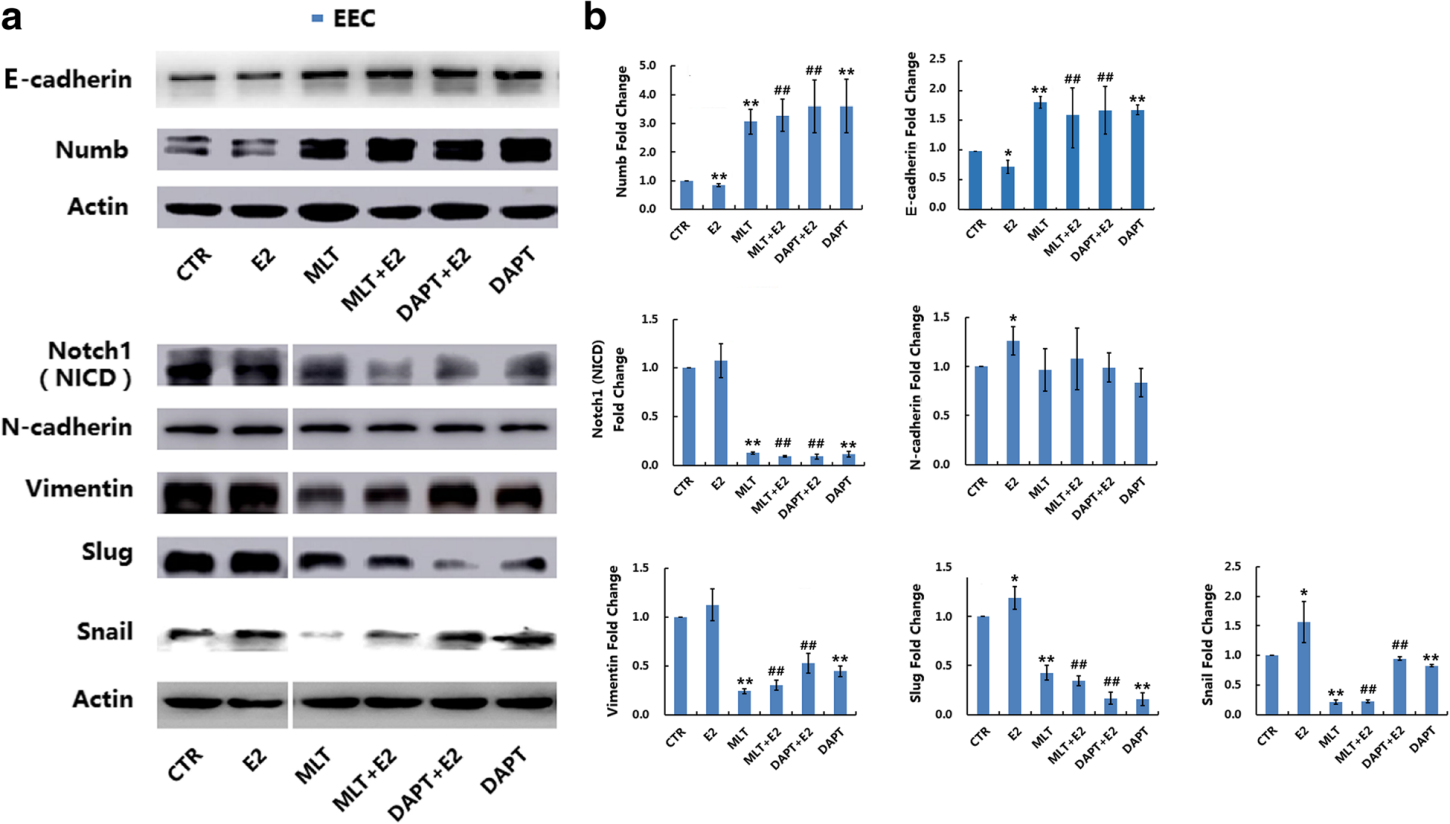
Melatonin reverses 17β-estradiol-induced EMT in EEC involving Notch1 signaling pathway. EEC were treated with different drugs for 72 h. **a** The protein expression levels of Notch1 (NICD), E-cadherin, N-cadherin, Vimentin, Slug, Snail, Numb and Actin were determined by western blot. β-actin was used as a loading control. **b** The ratios of Notch1/E-cadherin/N-cadherin/Vimentin/Slug/Snail/Numb to β-actin were analyzed. Data are presented as the mean ± SD. E2: 17β-estradiol; MLT: melatonin. *p < 0.05, ** *p* < 0.01 vs. untreated cells. #*p* < 0.05, ##*p* < 0.01 vs. E2-treated cells

**Fig. 6:**
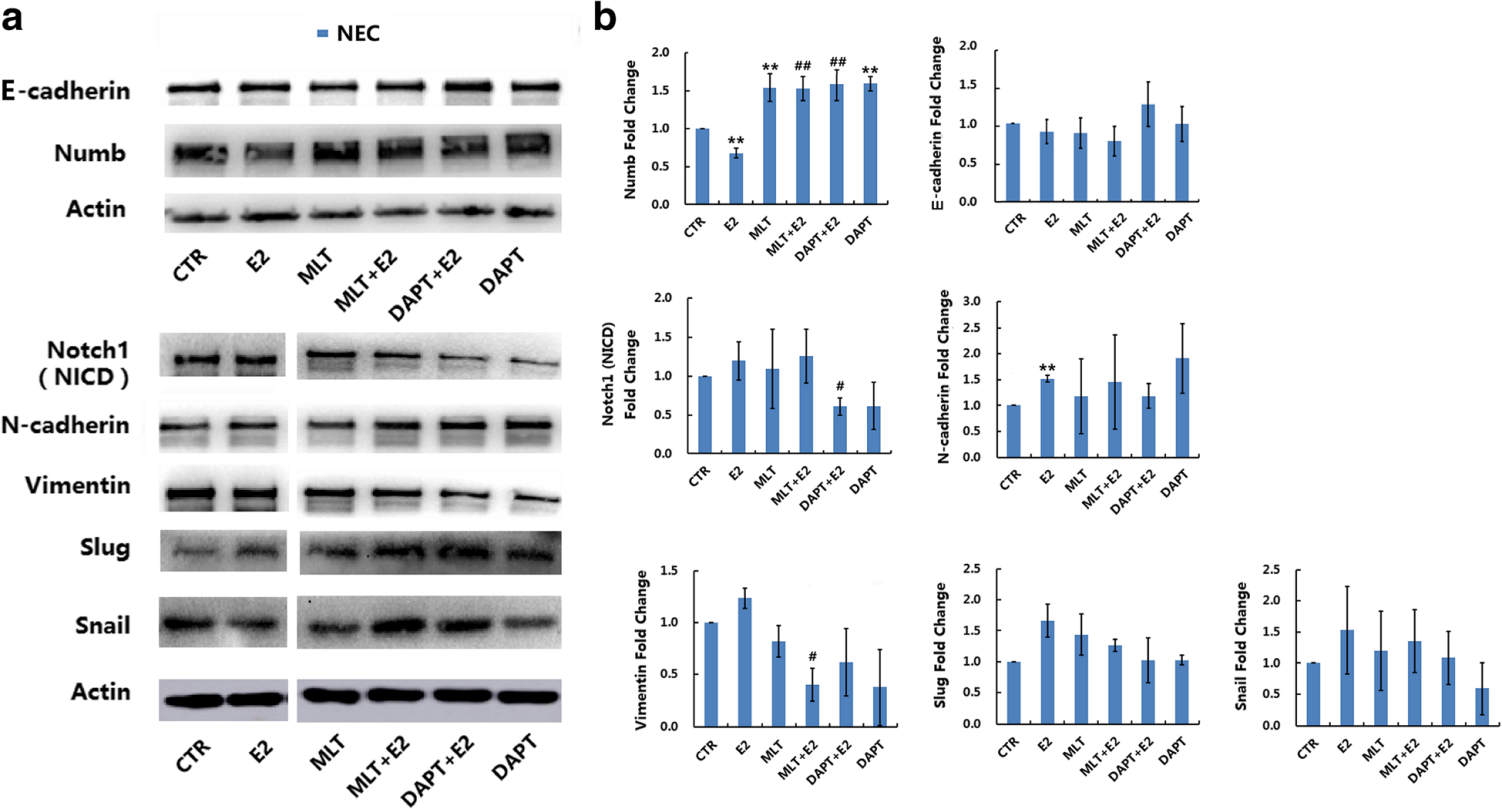
The expression of EMT and Notch signaling pathway-related markers in NEC. NEC were treated with different drugs for 72 h. **a** The protein expression of Notch1 (NICD), E-cadherin, N-cadherin, Vimentin, Slug, Snail, Numb and Actin were determined by western blot. β-actin was used as a loading control. **b** The ratios of Notch1/ E-cadherin/ N-cadherin/Vimentin/Slug/ Snail/Numb to β-actin were analyzed. Data are presented as the mean ± SD. E2: 17β-estradiol; MLT: melatonin. *p < 0.05, ** *p* < 0.01 vs. untreated cells. #*p* < 0.05, ##*p* < 0.01 vs. E2-treated cells

The expression of EMT markers was determined using western blot analysis. As shown in Fig. 5, 17β-estradiol significantly increased the expression of N-cadherin, Slug and Snail, and decreased the expression of Numb and E-cadherin in EEC (*p* < 0.05). In NEC, 17β-estradiol significantly increased the expression of N-cadherin and decreased the expression of Numb (Fig. 6, *p* < 0.05), but showed no significant effect on the expression of Notch1 (NICD), Vimentin, E-cadherin, Slug, or Snail (Fig. 6, *p* > 0.05). In EEC, DAPT significantly decreased the expression of Notch1 (NICD), Vimentin, Slug, and Snail and increased the expression of Numb and E-cadherin (Fig. 5, *p* < 0.05). DAPT also abolished 17β-estradiol-induced expression of Notch1 (NICD), Vimentin, Slug, and Snail and the downregulation of Numb and E-cadherin (Fig. 5, *p* < 0.05). In NEC, Notch inhibition by DAPT significantly increased the expression of Numb, and 17β-estradiol-caused downregulation of Numb (Fig. 6, *p* < 0.05).

In western blot analyses, melatonin significantly decreased the expression of Notch1 (NICD), Vimentin, Slug, and Snail and increased the expression of Numb and E-cadherin in EEC (Fig. 5, *p* < 0.05). Melatonin also abolished the 17β-estradiol-induced expression of Notch1 (NICD), Vimentin, Slug, and Snail and the downregulation of Numb and E-cadherin in EEC (Fig. 5, *p* < 0.05). In NEC, melatonin significantly increased the expression of Numb (Fig. 6, *p* < 0.05) but had no significant effect on the expression of Notch1 (NICD), Vimentin, N-cadherin, E-cadherin, Slug or Snail. Melatonin also abolished the 17β-estradiol-induced expression of Vimentin and downregulation of Numb in NEC (Fig. 6, *p* < 0.05).

**Fig. 7:**
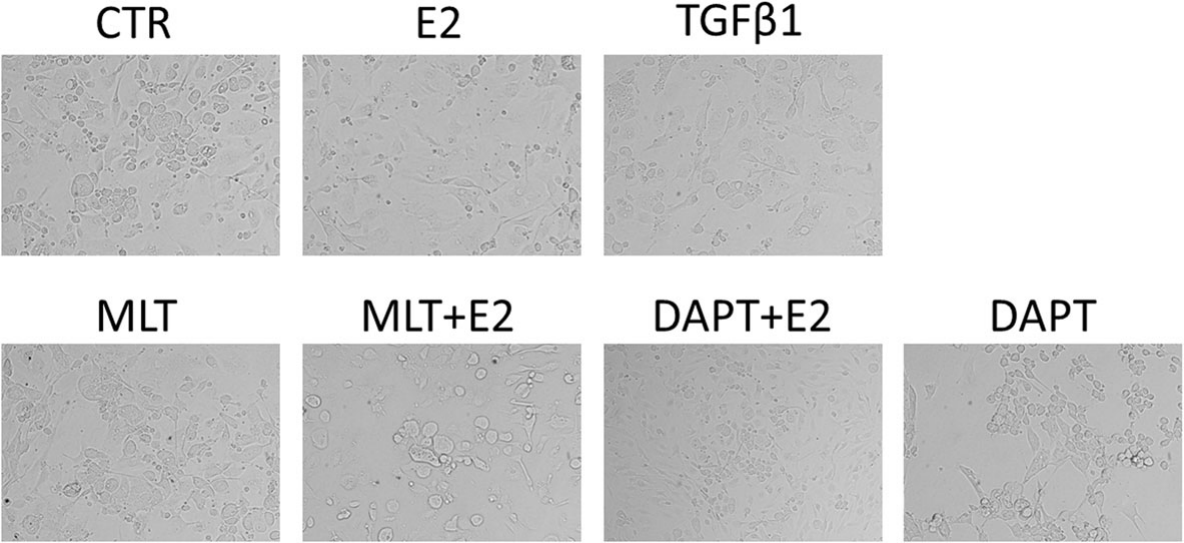
The morphology of EEC treated with E2, TGF-β1, Melatonin, DAPT, a combination of E2 and Melatonin, or a combination of E2 and DAPT for 48 h. The cells were observed using phase contrast microscopy at 400× magnification. Scale bar: 20 μm.E2: 17β-estradiol; MLT: Melatonin

**Fig. 8:**
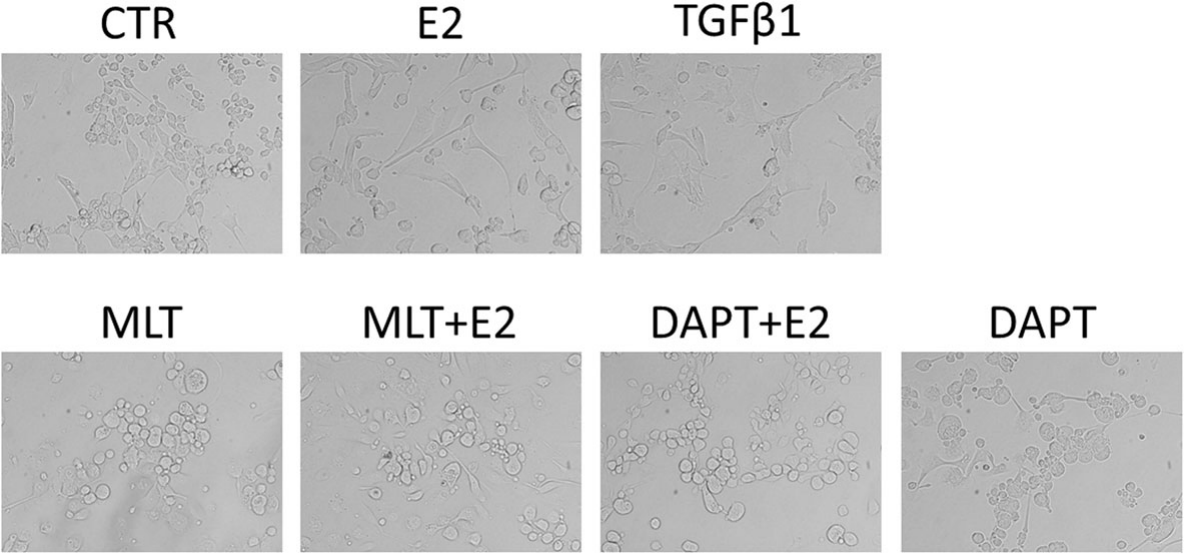
The morphology of NEC treated with E2, TGF-β1, Melatonin, DAPT, a combination of E2 and Melatonin, or a combination of E2 and DAPT for 48 h. The cells were observed using phase contrast microscopy at 400× magnification. Scale bar: 20 μm.E2: 17β-estradiol; MLT: Melatonin

We then examined the effect of 17β-estradiol and melatonin on the morphology of normal and endometriotic epithelial cells. The cells were treated with recombinant transforming growth factor-β1 (TGF-β1), which is known to be an EMT inducer. As expected, after stimulation with 0.78 nM of recombinant TGF-β1 for 48 h, both EEC (Fig. 7) and NEC (Fig. 8) showed reduction of the epithelial “cobblestone” morphology, became scattered, acquired a spindle-shaped morphology, and lost cell-cell contacts, all of which are characteristics of a mesenchymal-like morphology. 17β-estradiol exhibited similar effects as TGF-β1 in the cells. Treatment with 1 mM melatonin or 10 μM DAPT for 48 h abolished the TGF-β1 or 17β-estradiol-induced morphological changes in EEC and NEC.

## Discussion

Although endometriosis is a benign disease, it exhibits a series of biological behaviors similar to malignant tumors, including adhesion, invasion, and implantation [28]. In the current study, we found that aberrant expression of EMT-related markers existed in endometriotic eutopic endometrium, and estrogen promoted the migration, invasion and EMT phenotype in normal and endometritic eutopic epithelial cells, while melatonin and the blocking of Notch signaling inhibited 17β-estradiol-induced migration, invasion and epithelial-mesenchymal transition in normal and endometriotic endometrial epithelial cells.

The Notch signaling pathway is thought to be critical for the induction of EMT and is involved in the progression of a series of diseases [29, 30]. Notch signaling can promote TGF-β1-induced EMT via the induction of Snai1 [30]. Jagged1-mediated Notch signaling activation can elevate the expression of Snail and Slug, resulting in the repression of E-cadherin in various disease models [29]. Numb is an inhibitory regulator of Notch1 signaling that acts by promoting the ubiquitination and degradation of the Notch1 intracellular domain [31]. In the current study, decreased epithelial marker expression and elevated mesenchymal marker expression indicate that the phenotype of EMT exists in endometriosis. In addition, elevated expression of EMT inducer, Snail and Slug, was also noted in endometriotic eutopic endometrium, suggesting an essential role of EMT in the development and pathogenesis of endometriosis. Immunohistochemistry analysis also showed increased Notch1 (NICD) expression and decreased Numb expression in endometriotic eutopic epithelial cells, indicating that Notch1/Numb signaling might be involved in the regulation of EMT in the endometriotic eutopic endometrium. Furthermore, there is no significant difference between proliferative and secretory phases observed in endometriosis. In our previous studies, we found that the expression of Numb, Slug and E-cadherin showed no significant difference, but Snail, N-cadherin and Notch1 showed cycle changement between proliferative and secretory phases in normal endometria. This might be due to the aberrant level of hormones in endometriosis.

Vimentin is a mesenchymal cell marker and is increased in cells that undergo EMT as known. In immunochemistry assays, cytokeratin immunostaining is positive in normal and endometriotic epithelial cells, while Vimentin immunostaining was negative [20]. In western blot experiments, we found that Vimentin showed bands in both cells and showed differences in drug intervention. Then, we searched other literature and found that Vimentin was also determined in endometrial epithelial cells by western blot [32, 33]. We cannot explain this phenomenon and will continue to explore it in follow-up studies.

Endometriosis is an estrogen-dependent disease [34]. Considerable biochemical evidence demonstrates that aromatase activity and P450 aromatase mRNA expression were noted in endometrial tissues from endometriosis [35], suggesting that endometriotic tissues are able to produce estrogens locally. It has been reported that estrogen receptor (ERα) signaling regulates E-cadherin and EMT through slug, and estrogen is proved to be involved in the process of EMT [36]. In human ovarian and breast cancer cells, 17β-estradiol can induce EMT via the activation of the PI3K/AKT pathway by enhancing the expression of snail and slug [37, 38]. In prostate epithelium, the ERα-mediated enhanced estrogenic effect is a crucial inductive factor of epithelial dedifferentiation, giving rise to the activation of an EMT program [39]. However, the role of estrogen in the EMT of endometriosis is rarely reported. In the current study, we found that estrogen promoted the migration, invasion and mesenchymal phenotype in normal and endometritic eutopic epithelial cells, suggesting that estrogen plays a role in the regulation of EMT in endometrial epithelial cells. What’s more, 17β-estradiol activated the Notch pathway, which is a key signaling pathway in EMT regulation, and downregulated the expression of Numb, an inhibitory regulator of Notch signaling. In addition, DAPT, a specific Notch inhibitor, abolished the effect of 17β-estradiol in endometrial epithelial cells, suggesting that Notch signaling might participate in the effect of 17β-estradiol on migration, invasion and EMT-related markers.

Reversing the migration and invasion of eutopic endometrium should be meaningful for the prevention and treatment of endometriosis. In in-vivo studies, melatonin showed potential therapeutic effects on endometriosis in animal models [40–42]. However, the molecular mechanism of the melatonin effect remains unclear. In animal studies, melatonin significantly inhibits ovarian aromatase expression and increases the levels of uterine ERα and progesterone receptor [43]. The genotoxic changes in the uterus caused by estrogen might be prevented by giving melatonin [44]. The addition of melatonin to estrogen replacement treatment is associated with a decrease in endometrial proliferation and prevents the appearance of cellular atypia [45]. In non-photoperiodic animals such as rats, melatonin positively affects the endometrial morphology and improves embryo implantation [46]. These data indicate that the effect of 17β-estradiol can be modified by melatonin. In the current study, melatonin abolished the 17β-estradiol-induced proliferation, migration, invasion and EMT phenotype in both normal and endometriotic epithelial cells, demonstrating the protective effect of melatonin on the endometrium, especially on endometriotic endometrium, which has locally elevated estrogen levels [35].

In the current in-vitro study, melatonin showed a similar effect on normal and endomotriotic epithelial cells. However, in the local high estrogen microenvironment of endometriosis in vivo, melatonin may show anti-estrogen effects and therefore has a potential therapeutic effect.

Notch signaling pathway is a key signaling pathway for EMT regulation. Here, we found that Notch signaling pathway is also involved in EMT regulation in endometriosis. We showed that a specific inhibitor of Notch signaling pathway inhibited the proliferation, migration, invasion and in endometriotic epithelial cells and normal endometrial cells, but EMT-like phenotype was not inhibited in normal endometrial cells. The data suggested that Notch signaling plays a key role in the regulation of EMT in endometriosis. Melatonin can inhibit the activity of Notch1 signaling pathway in endometriotic epithelial cells, which is reflected in the decrease of NICD expression. This phenomenon was absent in normal endometrial epithelial cells. There is insufficient evidence that the Notch signaling pathway is a direct pathway for melatonin action or that it is the only downstream pathway for melatonin action in endometriosis. However, the data suggest that Notch signaling pathway may be a potential therapeutic target in endometriosis.

Studies have reported that Numb completely prevents EMT by antagonizing Notch signaling [47, 48]. In the present study, Melatonin and Notch-specific inhibitor promotes the expression of Numb in both normal and endometriotic endometrium, indicating that the effect of melatonin on Notch signaling might be mediated by Numb upregulation. We also proved that Numb expression was decreased in endometrium of endometriosis, implying that Numb might be a potent therapeutic target in endometriosis.

## Conclusions

In summary, we confirm that aberrant expression of Notch1/Numb signaling and an active EMT process are present in eutopic endometrium of endometriosis, and we provide an experimental basis for considering melatonin as a potential treatment for endometriosis. In addition, we observed that Notch signaling pathway might be involved in the progression of endometriosis. The role of Notch signaling pathway in the effect of estrogen and melatonin in endometriosis need further investigation in future studies.

## Abbreviations

CCK-8: Cell Counting Kit-8
EEC: Endometriotic eutopic epithelial cells
EMT: Epithelial-mesenchymal transition
ERα: Estrogen receptor alpha
FBS: Charcoal-stripped fetal bovine serum
MMPs: Matrix metalloproteinases
NEC: Normal endometrial epithelial cells
NFκB: Nuclear factor-κB
NICD: Notch1 intracellular domain
NSC: Normal stromal cells
OD: Optical density
TLR: Toll-like receptor
